# PTEN Inhibitor Treatment Lowers Muscle Plasma Membrane Damage and Enhances Muscle ECM Homeostasis after High-Intensity Eccentric Exercise in Mice

**DOI:** 10.3390/ijms24129954

**Published:** 2023-06-09

**Authors:** Baile Wu, Lijun Shi, Ying Wu

**Affiliations:** 1 Department of Exercise Physiology, School of Sports Science, Beijing Sport University, Beijing 100084, China; 2020210314@bsu.edu.cn (B.W.); shilj@bsu.edu.cn (L.S.); 2 Key Laboratory of Sports and Physical Health of the Ministry of Education, Beijing Sport University, Beijing 100084, China

**Keywords:** exercise-induced muscle damage1, VO-OHpic2, PTEN3, muscle extracellular matrix4, muscle membranes5

## Abstract

Exercise-induced muscle damage (EIMD) is a common occurrence in athletes and can lead to delayed onset muscle soreness, reduced athletic performance, and an increased risk of secondary injury. EIMD is a complex process involving oxidative stress, inflammation, and various cellular signaling pathways. Timely and effective repair of the extracellular matrix (ECM) and plasma membrane (PM) damage is critical for recovery from EIMD. Recent studies have shown that the targeted inhibition of phosphatase and tension homolog (PTEN) in skeletal muscles can enhance the ECM environment and reduce membrane damage in Duchenne muscular dystrophy (DMD) mice. However, the effects of PTEN inhibition on EIMD are unknown. Therefore, the present study aimed to investigate the potential therapeutic effects of VO-OHpic (VO), a PTEN inhibitor, on EIMD symptoms and underlying mechanisms. Our findings indicate that VO treatment effectively enhances skeletal muscle function and reduces strength loss during EIMD by upregulating membrane repair signals related to MG53 and ECM repair signals related to the tissue inhibitor of metalloproteinases (TIMPs) and matrix metalloproteinase (MMPs). These results highlight the potential of pharmacological PTEN inhibition as a promising therapeutic approach for EIMD.

## 1. Introduction

Exercise-induced muscle damage (EIMD) is a common physiological response to various types of exercise and is characterized by the histological evidence of plasma membrane (PM) and extracellular matrix (ECM) structure disruption. This damage can lead to delayed onset muscle soreness (DOMS), reduced athletic performance, and an increased risk of secondary injury [1]. EIMD involves oxidative stress, inflammation, and various cellular signaling pathways [2]. The severity of EIMD is dependent on the type, intensity, and duration of exercise, as well as the individual’s fitness level and recovery ability [3]. Understanding the mechanisms underlying EIMD and developing effective strategies to prevent or mitigate its negative effects are crucial for athletes, coaches, and trainers. Therefore, EIMD has been a topic of considerable interest in the fields of sports science and exercise physiology, with numerous studies investigating its pathophysiology, diagnosis, prevention, and treatment [3,4,5].

The disruption of the PM [6,7,8] and ECM [9,10] of skeletal muscle fibers is a major contributor to the development of EIMD. The severity and repair process of EIMD-induced skeletal muscle PM and ECM damage can vary depending on various factors, including the type, intensity, and duration of exercise, as well as the individual’s fitness level and recovery ability [5]. Chronic PM and ECM degradation due to EIMD have been shown to negatively impact athletic performance [11,12,13], highlighting the importance of identifying the underlying causes of EIMD and promoting timely and effective repair of the PM and ECM after exercise-induced damage.

Ensuring the prompt and efficient repair of damage to both the PM and ECM following exercise-induced stress is essential [14]. The ECM provides structural support to the muscle fibers and is essential for force transmission and maintenance of tissue integrity [15]. Damage to the ECM leads to disruption of these functions, which can compromise athletic performance and increase the risk of secondary injury. Similarly, PM damage can result in loss of muscle function and increased vulnerability to further damage [16]. The repair of ECM and PM damage is a complex process involving multiple cellular and molecular mechanisms, including the activation of signaling pathways and the recruitment of repair proteins [16,17]. EIMD causes chronic negative effects on athletic performance due to membrane and ECM degradation. Effective repair of ECM and PM damage after EIMD can prevent further damage, reduce recovery time, and improve athletic performance. Therefore, understanding the mechanisms underlying ECM and PM repair and developing strategies to enhance these processes are crucial for athletes, coaches, and trainers.

Phosphatase and tension homolog (PTEN) is a crucial tumor suppressor gene and negative regulator of skeletal muscle hypertrophy [18,19,20]. Its function is primarily associated with the PI3K/Akt/mTOR signaling pathway [20], which plays a critical role in cell growth [21], proliferation [22], and survival [23]. In recent research focusing on Duchenne muscular dystrophy (DMD), a genetic disorder characterized by progressive muscle degeneration, it has been observed that PTEN expression is elevated in DMD mice [24]. Interestingly, studies have shown that the targeted knockout of PTEN in skeletal muscles of DMD mice can lead to significant improvements in muscle health [24]. Specifically, PTEN knockout promotes the simultaneous repair of the skeletal muscle PM and ECM, which are vital for maintaining muscle integrity and function [24]. This repair process is mediated by the upregulation of the key membrane repair protein MG53 and the modulation of ECM-related proteins [24]. Furthermore, the use of a potent PTEN inhibitor called VO-OHpic (VO) has shown promising results in treating ECM and PM damage in skeletal muscles [24]. This inhibitor effectively enhances the recovery of ECM and PM damage, indicating its potential as a therapeutic intervention for muscle-related conditions. It is important to note that frequent damage to the PM and ECM is a hallmark of DMD and EIMD [16,25,26]. The impaired motor function observed in DMD mice is closely associated with this damage. Similarly, during EIMD, the loss of muscle function is largely attributed to PM and ECM damage [16,25,26]. We have observed the significant regulatory potential of PTEN in PM and ECM repair. However, whether PTEN inhibition similarly regulates skeletal muscle function during EIMD remains unknown. Therefore, the aim of this study was to investigate whether the pharmacological PTEN inhibitor, VO, modulates muscle function during EIMD and to explore the potential underlying mechanisms. Our findings demonstrated that VO treatment effectively enhanced skeletal muscle function and reduced strength loss during EIMD. These improvements were attributed to the upregulation of membrane repair signals mediated by MG53 and ECM repair signals involving the tissue inhibitor of metalloproteinases (TIMPs) and matrix metalloproteinases (MMPs). Overall, our research provides valuable insights into the diagnosis, prevention, and nutritional strategies for EIMD. It highlights the potential of pharmacological PTEN inhibition as a promising therapeutic approach for improving muscle function and promoting recovery from PM and ECM damage.

## 2. Results

### 2.1. Two-Week VO-OHpic Treatment Improves Motor Function during EIMD in Mice

**Figure 1:**
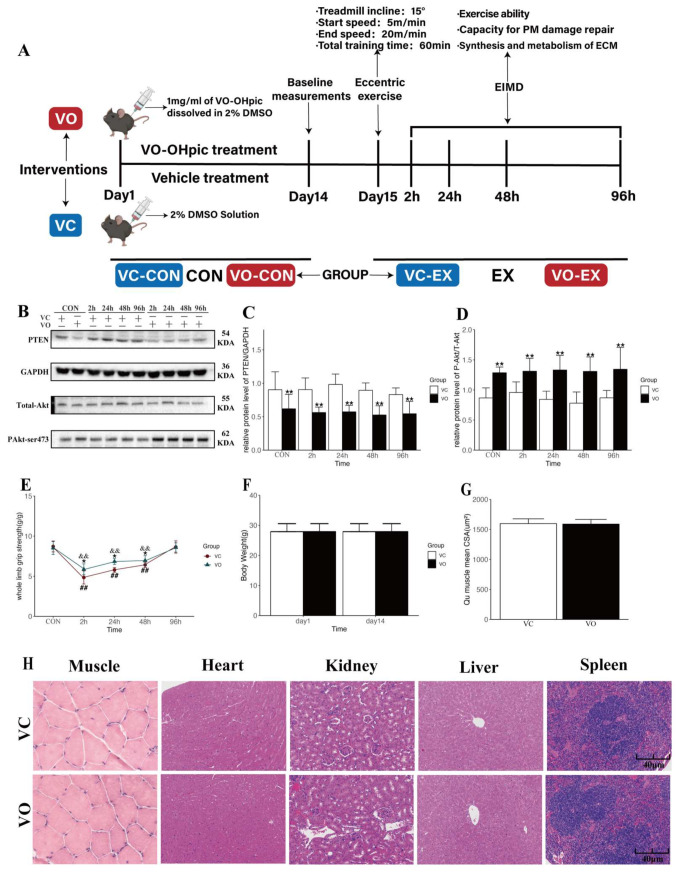
Experimental design and effects of VO treatment on muscle function and safety assessment in mice. (**A**) Schematic representation of the experimental design. (**B**) Representative Western blot images of PTEN, Akt, and pAkt protein expression. (**C**) Quantification of PTEN protein expression normalized to GAPDH. (**D**) Quantification of pAkt/total-Akt ratio. (**E**) Effect of VO on recovery of whole-limb grip strength after eccentric EIMD in mice. (**F**) Effect of two-week VO intervention on body weight in mice. (**G**) Effect of two-week VO intervention on the CSA of the Qu muscle in mice. (**H**) Representative images of HE-stained sections of mouse muscle, heart, kidney, and liver tissues. Scale bar = 40 μm. *n* = 3 mice per group. All data are presented as mean ± standard deviation (mean ± SD). * *p* < 0.05, ** *p* < 0.01 between VC and VO. ## *p* < 0.01 vs. VC-CON. && *p* < 0.01 vs. VO-CON.

The experimental design of this study is shown in Figure 1A. The mice were treated with the PTEN-specific pharmacological inhibitor, VO [27,28], or its vehicle control (VC) for 14 days to establish the PTEN pharmacological inhibition model. To confirm the effectiveness of the PTEN pharmacological inhibition model, we examined the protein expression of PTEN, Akt, and the pAkt/total Akt ratio in the quadriceps (Qu) muscle of mice. Our results showed that compared to the VC group, Qu muscle PTEN protein expression was significantly lower in mice treated with VO for two weeks (Figure 1B,C), with a higher pAkt/total Akt ratio (Figure 1B,D), indicating the successful establishment of the PTEN pharmacological inhibition model.

The mice underwent high-intensity eccentric exercise 24 h after 14 days of VO or VC treatment to induce EIMD (Figure 1A). The study aimed to investigate the impact of VO treatment on exercise performance during the EIMD period and explore the underlying mechanisms. Whole-limb grip strength (WGS) was measured at 2 h, 24 h, 48 h, and 96 h after exercise in both VO-EX and VC-EX groups (Figure 1E). The results showed significant declines in WGS at 2 h, 24 h, and 48 h after exercise in both groups (Figure 1E). However, the VO-EX group exhibited a smaller strength decrease and faster recovery in muscle strength induced by EIMD, compared to the VC-EX group (Figure 1E). These findings demonstrate that VO treatment attenuated acute declines in skeletal muscle strength caused by EIMD and facilitated the early recovery of muscle function.

The PTEN signaling pathway is known to act as a negative regulator of skeletal muscle hypertrophy, while PTEN inhibition promotes skeletal muscle fiber hypertrophy [19]. To investigate whether PTEN inhibition induces skeletal muscle fiber hypertrophy, we measured the body weight and mean cross-sectional area (CSA) of Qu muscles in mice treated with VO for two weeks. Our results indicated no significant difference in body weight (Figure 1F) and Qu muscle CSA (Figure 1G) between the VO and VC groups. These findings suggest that the VO treatment was insufficient to induce skeletal muscle hypertrophy and that the attenuation of VO-induced WGS decrease was not due to muscle fiber hypertrophy.

To ensure that the VO used in this study was safe and effective in mice in terms of intervention dose, frequency, and duration, and did not cause any potential adverse effects on skeletal muscle and internal organs, we recorded changes in visceral morphology and organ indices (heart, kidney, pancreas, liver, and skeletal muscle) in mice after two weeks of VO intervention (Figure 1H, Table 1). The results showed no significant changes in organ indices in mice treated with VO compared to the VC group. Additionally, histological examination of various organs using hematoxylin and eosin (HE) staining revealed no significant pathological changes in the VO group compared to the VC group. The results suggested that VO treatment did not cause any significant adverse effects on the visceral morphology and organ indices in mice.

### 2.2. PTEN Inhibition Enhances Myofibrillar Membrane Repair during EIMD

**Figure 2:**
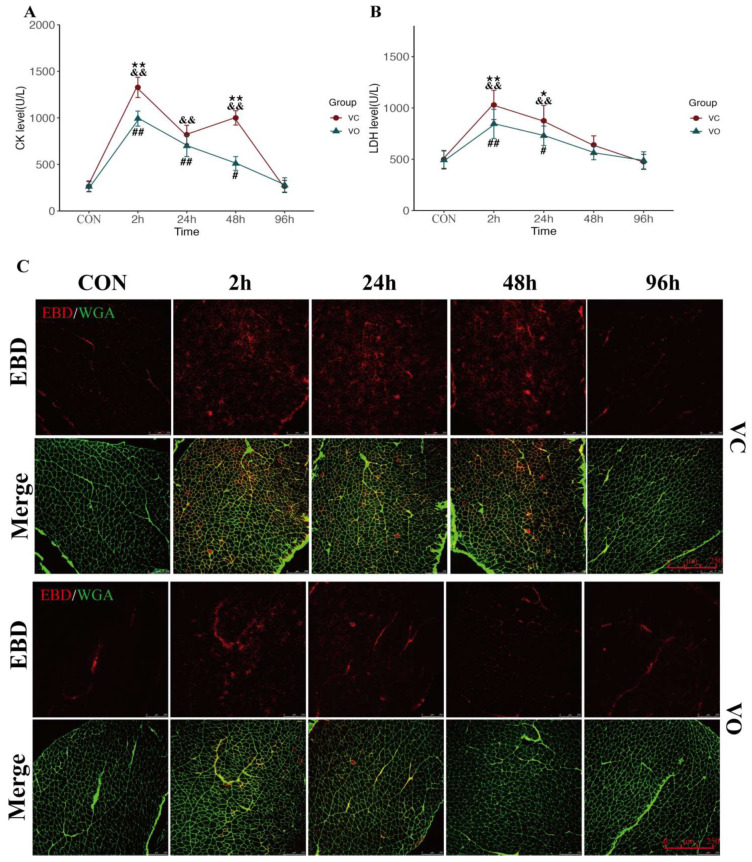
PTEN inhibition enhances myofibrillar membrane repair during EIMD. (**A**) The effect of VO on serum CK levels in EIMD mice. (**B**) The effect of VO on serum LDH levels in EIMD mice. (**C**) Representative immunofluorescence images of EBD-positive cell areas on cross-sections of Qu muscles; EBD is represented by red fluorescence, while WGA is represented by green fluorescence, scale bar = 250 μm, *n* = 3. All data are presented as mean ± SD. * *p* < 0.05, ** *p* < 0.01 between VC and VO. # *p* < 0.05, ## *p* < 0.01 vs. VC-CON. && *p* < 0.01 vs. VO-CON.

The disruption of muscle membrane integrity during eccentric exercise is a characteristic hallmark of EIMD [29]. In this study, we aimed to determine the effect of VO treatment on muscle PM integrity following EIMD. We assessed serum levels of CK and LDH, markers of muscle injury, in mice after EIMD. Our results showed that the CK levels in both the VC-EX and VO-EX groups were significantly elevated at 2~48 h post-exercise (Figure 2A), and the LDH levels were significantly higher at 2~24 h post-exercise (Figure 2B). However, the CK levels in the VO-EX group were significantly lower than those in the VC-EX group at 2~48 h, and the LDH levels in the VO-EX group were significantly lower at 2~24 h post-exercise (Figure 2A). Moreover, the CK levels in the VC-EX group exhibited two peaks at 2 h and 48 h post-exercise, while the CK levels in the VO-EX group showed a continuous decline (Figure 2A), indicating that secondary injury may have occurred to muscle PM in the VC-EX group during EIMD.

**Figure 3:**
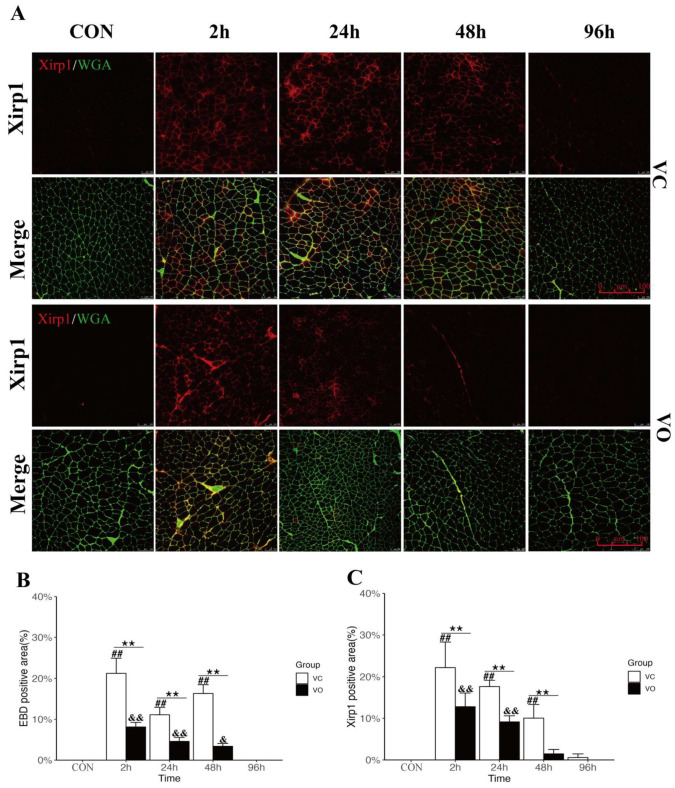
PTEN inhibition enhances myofibrillar membrane repair during EIMD. (**A**) Representative immunofluorescence images of the membrane damage marker Xirp1. Xirp1 is represented by red fluorescence, while WGA is represented by green fluorescence, scale bar = 100 μm, *n* = 3. (**B**) Quantitative analysis of EBD positive area. (**C**) Quantitative analysis of Xirp1 positive area. All data are presented as mean ± SD. ** *p* < 0.01 between VC and VO. ## *p* < 0.01 vs. VC-CON. & *p* < 0.05, && *p* < 0.01 vs. VO-CON.

To further examine the effect of VO treatment on skeletal muscle PM repair, we evaluated the changes in Evans blue dye (EBD) uptake, a marker of skeletal muscle PM injury, during EIMD. Muscle PM injury is often accompanied by cytoskeletal depolymerization during EIMD [30]; hence, we also assessed the changes in Xin-actin-binding repeat-containing protein1 (Xirp1), a marker of cytoskeletal injury, during EIMD. We labeled the muscle PM using wheat germ agglutinin (WGA) and evaluated the effects of VO on skeletal muscle PM and cytoskeletal injury by detecting the fluorescence expression of EBD and Xirp1 in the cross-sections of the Qu muscle using laser scanning confocal microscopy. The percentage of EBD and Xirp1-positive cell areas was used to assess the effect of VO on skeletal muscle PM and cytoskeletal injury. The immunofluorescence results demonstrated that EBD levels in the Qu muscle of the VC-EX mice exhibited a bimodal effect like CK, with two peaks at 2 h and 48 h post-exercise (Figure 2C and Figure 3B), indicating secondary skeletal muscle injury during EIMD. On the other hand, Xirp1 levels in the VC-EX group peaked only at 2 h post-exercise (Figure 3A,B), lasted until 48 h, and returned to VC-CON levels at 96 h. In contrast, the positive regions of EBD and Xirp1 in the Qu muscle of VO-EX mice were significantly lower than those in the control group at 2 h, 24 h, and 48 h during EIMD, and no secondary elevation of EBD was observed in the VO-EX group.

These findings suggest that VO treatment reduces skeletal muscle PM damage during EIMD by mitigating cytoskeletal depolymerization and decreasing the exchange of substances between intracellular and extracellular spaces, which may contribute to alleviating inflammation, oxidative stress, and impairment of cellular signaling pathways.

### 2.3. PTEN Inhibition Upregulates MG53 to Promote Muscle Membrane Repair

**Figure 4:**
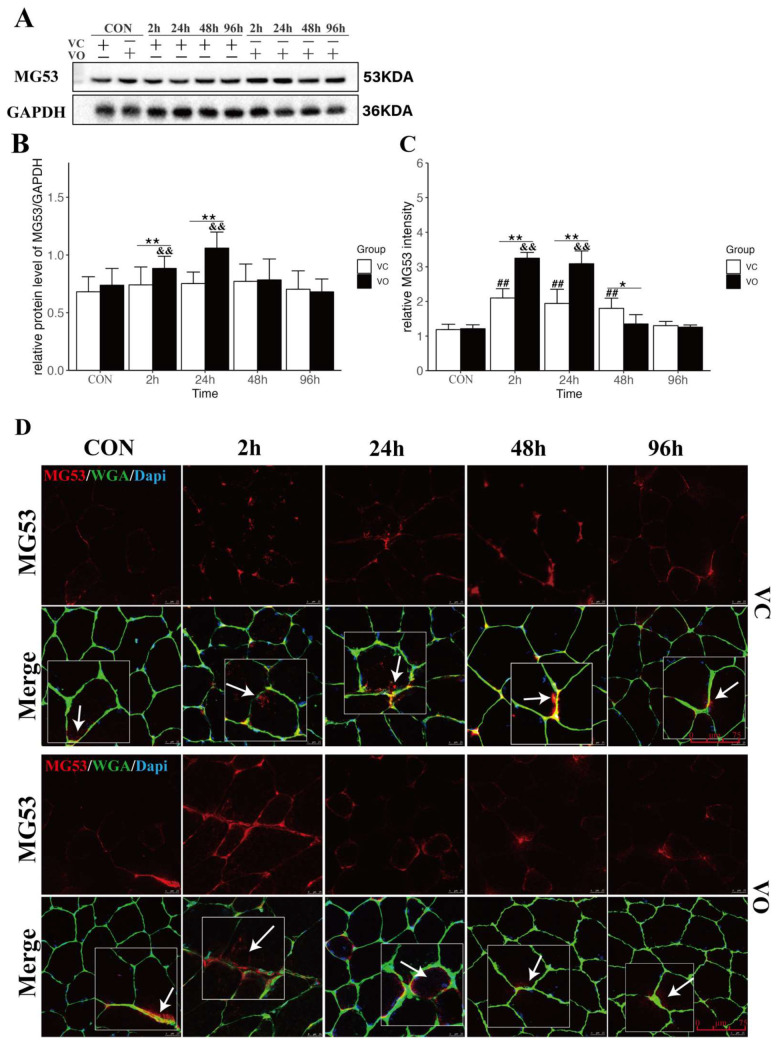
PTEN inhibition upregulates MG53 to promote muscle membrane repair. (**A**) Representative Western blot images of MG53 protein expression. (**B**) Quantification of MG53 protein expression normalized to GAPDH. (**C**) Quantitative analysis of the relative MG53 intensity. (**D**) Representative immunofluorescence images of the membrane repair signal MG53; MG53 is represented by red fluorescence, while WGA is represented by green fluorescence; DAPI is represented by green fluorescence; the arrow indicates the local enlargement of MG53, scale bar = 50 μm, *n* = 3. All data are presented as mean ± SD. * *p* < 0.05, ** *p* < 0.01 between VC and VO. ## *p* < 0.01 vs. VC-CON. && *p* < 0.01 vs. VO-CON.

To further investigate the underlying mechanisms by which VO promotes muscle membrane repair, we analyzed the protein expression (Figure 4A,B) of the membrane repair protein MG53 and conducted immunofluorescence localization in skeletal muscles (Figure 4C,D). Our results clearly demonstrated that in the VO-EX group, the levels of MG53 were significantly higher compared to the VO-CON and VC-EX groups at both 2 h and 24 h post-exercise (Figure 4A,B). This indicates that VO treatment effectively upregulates the expression of MG53 during the early stages of EIMD.

Immunofluorescence images of MG53 further revealed the accumulation of MG53 on the plasma membrane of skeletal muscles in both the VO-EX and VC-EX groups at 2 h after EIMD (Figure 4C,D), suggesting the essential role of MG53 in the early-stage repair of the skeletal muscle plasma membrane during EIMD. However, it is noteworthy that the fluorescence intensity of MG53 was significantly higher in the VO-EX group compared to the VC-EX group at both 2 h and 24 h after eccentric exercise (Figure 4C,D). Furthermore, as time progressed, the fluorescence intensity of MG53 in the VO-EX group returned to baseline levels by 48 h, whereas in the VC-EX group, it took 96 h to reach baseline levels (Figure 4C,D). These findings suggest that the VC-EX group experienced more persistent damage to the skeletal muscle plasma membrane during EIMD, implying that the physiological process of plasma membrane repair may endure for a longer duration. On the other hand, the elevated expression of MG53 induced by VO treatment facilitated the restoration of skeletal muscle plasma membrane integrity, highlighting the beneficial effects of VO in promoting muscle membrane repair during EIMD.

### 2.4. PTEN Inhibition Regulates the Synthesis and Catabolism of ECM during EIMD

Under physiological conditions, ECM metabolism and catabolism maintain a dynamic balance. However, this balance is disrupted by mechanical stress, such as that caused by eccentric exercise, which can lead to severe muscle pain and degradation of muscle ECM [12,31] In this study, we investigated the effect of VO on the ECM in a mouse model of EIMD.

Our preliminary observations using scanning electron microscopy (SEM) revealed more severe damage to the ECM microstructure in the VC-EX group during EIMD, characterized by the presence of visible gaps within the ECM (Figure S1). This observation suggests that VO treatment may influence the processes of ECM synthesis and degradation during EIMD.

**Figure 5:**
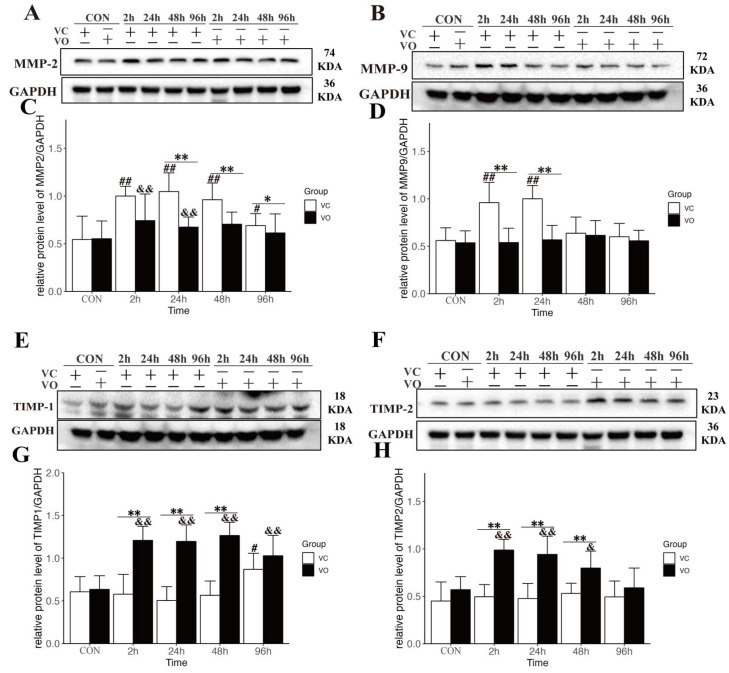
PTEN inhibition regulates the synthesis and catabolism of ECM during EIMD. (**A**) Representative Western blot images of MMP-2 protein expression. (**B**) Representative Western blot images of MMP-9 protein expression. (**C**) Quantification of MMP-2 protein expression normalized to GAPDH. (**D**) Quantification of MMP-9 protein expression normalized to GAPDH. (**E**) Representative Western blot images of TIMP-1 protein expression. (**F**) Representative Western blot images of TIMP-2 protein expression. (**G**) Quantification of TIMP-1 protein expression normalized to GAPDH. (**H**) Quantification of TIMP-2 protein expression normalized to GAPDH. All data are presented as mean ± SD. * *p* < 0.05, ** *p* < 0.01 between VC and VO. # *p* < 0.05, ## *p* < 0.01, vs. VC-CON. & *p* < 0.05, && *p* < 0.01 vs. VO-CON.

To further explore this hypothesis, we assessed the protein expression levels of MMP-2 (Figure 5A,C) and MMP-9 (Figure 5B,D), which are known to be involved in ECM degradation [9]. Our results showed that MMP-2 levels in the VO-EX group were significantly lower than those in the VC-EX group at 24–96 h post-exercise (Figure 5A,C). Similarly, MMP-9 levels in the VC-EX group were significantly higher at 2 h and 24 h post-exercise, while no significant differences in MMP-9 expression were observed between the VO-EX groups after exercise (Figure 5B,D). These findings indicate an elevation of MMP-2/9 levels after EIMD, while the PTEN inhibition mediated by VO may suppress excessive MMP expression. Additionally, we examined the protein expression levels of TIMP-1 (Figure 5E,G) and TIMP-2 (Figure 5F,H) during EIMD. Our results showed that TIMP-1 and TIMP-2 levels in the VO-EX group were significantly higher than those in the VC-EX group at 2–48 h post-exercise. This suggests an upregulation of TIMP-1/2 expression by VO, which may promote the inhibition of MMPs. Overall, these findings support the notion that VO treatment modulates the expression of key proteins involved in ECM degradation and synthesis, thereby influencing the balance between these processes.

## 3. Discussion

The goal of this study was to investigate the potential role of PTEN pharmacological inhibition in facilitating skeletal muscle recovery during EIMD, as well as to understand the underlying mechanisms. Our findings provide compelling evidence that PTEN inhibition enhances the restoration of exercise function during EIMD by concurrently ameliorating PM and ECM damage.

The PTEN gene plays a pivotal role in regulating vital cellular signaling pathways, which include PI3K/Akt/mTOR, FAK/p130cas, and ERK/MAPK [20,32,33]. It is primarily responsible for inhibiting cell adhesion and migration, inducing apoptosis, halting cell cycle progression and proliferation, inhibiting angiogenesis, aiding DNA repair, and managing normal embryonic development, aging, and metabolism, among other processes [23,24,34,35,36]. Earlier research has evaluated PTEN’s protective contribution to skeletal and cardiac muscle injuries [37,38,39]. In this study, we leveraged VO to construct a model of pharmacological PTEN inhibition. VO has demonstrated efficiency as a PTEN inhibitor, displaying high specificity for PTEN. It can inhibit PTEN even at low nanomolar concentrations [40] and importantly, this inhibitory effect is completely reversible [40]. While concerns have been raised regarding the potential tumorigenesis risks associated with systemic PTEN inhibition therapy [27], nevertheless, our investigation revealed that intervention with VO does not significantly affect visceral indices, nor does it induce observable morphological or pathological changes within various visceral organs. This observation underscores a gap in our understanding of the potential impact of VO on visceral organ function, constituting one of the limitations of the current study. Therefore, to establish a comprehensive safety and efficacy profile for VO and similar PTEN inhibitory therapies, it is imperative to conduct further longitudinal studies and rigorous examination of their potential impacts on visceral organ function and overall systemic health.

MG53, a member of the TRIM family of E3 ubiquitin ligases, also known as TRIM72, is specifically expressed in skeletal and cardiac muscle and is the only protein of the TRIM family involved in muscle membrane repair [41]. The membrane repair mechanisms of endogenous MG53 and exogenous MG53 differ. The interaction of endogenous MG53 with its binding partners Dysferlin [42] and Caveolin3 [42] is indispensable for cell membrane repair. However, the repair effect of exogenous MG53 is neither Dysferlin- nor Caveolin-dependent [43]. Given the specific role of exogenous MG53 in membrane repair, exogenous MG53 has been used to treat conditions linked to abnormalities in membrane repair, and numerous studies have demonstrated that exogenous MG53 has potent therapeutic potential for injuries to skeletal muscle [43], cardiac muscle [44], the liver [45], the lungs [46], and the kidneys [47]. Our study discerned that the application of VO notably reduces the levels of CK and LDH, which serve as serum indicators of skeletal muscle damage, in conjunction with the PM injury marker EBD during EIMD. Simultaneously, this treatment effectively amplifies the expression of MG53 protein within skeletal muscles throughout EIMD, implying the potential capability of VO to bolster MG53’s PMR functionality. In this study, we observed that the levels of CK and the percentage of EBD-positive cells in the VO-EX group did not show a second increase as they did in the VC-EX group. These results suggest that PTEN inhibition therapy might affect other potential pathways involved in the PMR process, such as inflammation and oxidative stress. Regrettably, while we observed this phenomenon, we did not delve into it in this study. Future investigations are needed to further clarify whether PTEN inhibition influences PMR through these pathways. Moreover, the significant upregulation of MG53 expression induced by VO treatment plays a crucial role in promoting PMR during the early stages of EIMD. In contrast, the VC-EX group shows no substantial changes in MG53 protein expression, and the fluorescence intensity persists until the later stages of EIMD. These findings suggest that the physiological process of PMR may persist for an extended period. Consequently, it implies that enhancing the PMR pathway represents a reliable and effective strategy for facilitating the recovery of muscle function in the context of EIMD.

Under normal physiological conditions, the ECM of skeletal muscle undergoes a dynamic process of synthesis and degradation, maintaining the balance necessary for proper muscle function [48]. However, when exposed to mechanical stress during eccentric exercise, this delicate balance is disrupted, resulting in significant degradation of the ECM [49]. This aberrant ECM remodeling is considered a critical factor contributing to the pain and discomfort experienced in EIMD [50]. MMP-2 and MMP-9, key proteins involved in the breakdown of ECM components, are important in ECM degradation and remodeling. Studies have consistently demonstrated that the increased expression and activity of MMP-2 and MMP-9 are closely associated with acute ECM degradation following eccentric exercise [31,51]. These proteases play a crucial role in the remodeling of skeletal muscle ECM, disrupting tissue structure and function.

Interestingly, previous research has shown that inhibiting PTEN can effectively reduce myocardial fibrosis and inhibit MMP activation [39]. Building upon these findings, our study sought to investigate the potential therapeutic effect of PTEN inhibition in ECM preservation during EIMD. Our findings revealed that VO treatment inhibited MMP-2/9 expression and activity in the context of EIMD. This effect was associated with an upregulation of TIMP-1/2 expression, proteins known to function as inhibitors of MMPs. TIMP-1/2 exerts its regulatory role by binding to and neutralizing the activity of MMP-2/9, preventing excessive ECM degradation. Thus, the observed increase in TIMP-1/2 expression suggests a protective mechanism against acute ECM degradation induced by eccentric exercise. Notably, we observed visible improvement in ECM in the VO-EX group during EIMD through scanning electron microscopy. However, it is important to note that our current study lacks a comprehensive quantitative analysis of ECM microstructural damage. Future research efforts should focus on developing reliable quantitative analysis systems that can provide a more detailed assessment of ECM microstructural alterations. Such advancements would greatly contribute to our understanding of skeletal muscle injuries and other ECM-related disorders.

In conclusion, our study highlights the detrimental effects of mechanical stress on muscle ECM and PM, which in turn negatively impact muscle function. Importantly, pharmacological PTEN inhibition using VO demonstrates promising effects by suppressing MMP-2/9 expression and promoting TIMP-1/2 expression, thereby enhancing ECM integrity. Additionally, the upregulation of MG53 expression contributes to the repair of PM damage induced by EIMD. However, it remains crucial to gain a deeper understanding of PTEN’s involvement in pathways related to skeletal muscle PMR, including inflammation, oxidative stress, and apoptosis. Moreover, further exploration of robust quantitative analysis methods to accurately assess changes in ECM microstructure will undoubtedly enhance our understanding of muscle injury and provide valuable insights for the development of targeted therapeutic strategies.

## 4. Materials and Methods

### 4.1. Animals

Male C57BL/8N mice with specific pathogen-free (SPF) grades, aged 8 weeks and purchased from Charles River Laboratories China, Inc. (Beijing, China), were provided with ad libitum access to standard rodent chow and water. They were housed in cages in the mouse breeding room at the Research Center of Beijing Sport University. The protocol was approved by the Animal Research Ethics Committee of Beijing Sport University (protocol code 2021057A and date of approval 22 September 2020).

### 4.2. EIMD Protocol

High-intensity exercise, specifically Armstrong’s eccentric exercise model [52], is known to induce EIMD. The EIMD protocol included three days of acclimation training followed by a single session of high-intensity eccentric exercise.

Acclimatization training: 3 days, 0° slope of the running platform, 10 m/min running speed, 10 min initial duration, once a day, in 5-min increments.

Formal training: two days of rest after acclimatization training, followed by high-intensity eccentric exercise with a treadmill incline of −15°, increasing by 1 m/min until 20 m/min, total training time 60 min.

### 4.3. PTEN Inhibitor Treatment

PTEN inhibition scheme modified from the study of Yue [24]. The PTEN inhibitor VO-OHpic trihydrate (VO) (MedChemExpress, South Brunswick Township, NJ, USA) was dissolved at a final concentration of 1 mg/mL in a 2% dimethyl sulfoxide (DMSO) (MedChemExpress, South Brunswick Township, NJ, USA) aqueous solution containing 40% PEG300 (MedChemExpress, South Brunswick Township, NJ, USA) and 2% Tween 80 (MedChemExpress, South Brunswick Township, NJ, USA). The vehicle (VC) (2% DMSO solution containing 40% PEG300 and 2% Tween 80) was injected intraperitoneally into 8-week-old mice at a dose of 10 mg/kg body weight for 14 consecutive days, while VC was injected intraperitoneally into the control mice.

### 4.4. Animals Experiments

The study employed a randomized experimental design; to ensure sufficient statistical power in this study, sample size calculation was performed using G*Power software (Version 3.1.9.6). The specified parameters were set as follows: alpha level of 0.05, effect size of 0.4, and power of 0.8. This calculation was conducted to determine the appropriate sample size and ensure that the study would have adequate statistical power, with all animals being allocated to one of four groups: the pre-exercise control group treated with VC (VC-CON; *n* = 12), the pre-exercise control group treated with VO (VO-CON; *n* = 12), the exercise group treated with VC (VC-EX; *n* = 48), and the exercise group treated with VO (VO-EX; *n* = 48). After 12 h of completing the VO or VC interventions, 8 mice from each CON group were sacrificed, and samples of visceral organs, skeletal muscle, and blood were collected for the evaluation of VO safety effects and to serve as baseline values for subsequent analysis in this study. The remaining mice underwent functional tests, and after the completion of the tests, they were euthanized. At each time point post-exercise (2 h, 24 h, 48 h, and 96 h), 8 mice from each exercise group were sacrificed, while the remaining mice were subjected to functional testing. Skeletal muscle and blood samples were collected at each time point to facilitate subsequent analyses. All mice were anesthetized with a 15% concentration of Urethane at a dose of 2.5 mL/kg body weight prior to euthanasia. After the blood samples were collected, the mice were euthanized by cervical dislocation. The euthanasia procedure for mice has been approved by the Ethics Committee of Beijing Sport University (protocol code 2021057A and date of approval 22 September 2020).

### 4.5. Whole-Limb Grip Strength Test

The grip strength of mice was evaluated using a digital grip meter (Tarzan Technology Co., Shanghai, China). Each mouse was placed on the grip meter, allowing its paws to grasp the metal pull bar. Then, the mouse’s tail was gently pulled back until it lost grip on the bar, and the peak tension at release was recorded as the grip strength measurement. To ensure accuracy and reliability, each mouse was subjected to this procedure three times, and the average of the three measurements was used for analysis. To standardize the grip strength measurements, the values were normalized based on the body weight of each mouse. This was achieved by dividing the peak tension (g) by the body weight of the mouse (g). The resulting value was expressed as grams per gram (g/g) of body weight.

### 4.6. CK and LDH Level Test

Serum was collected from the medial canthal vein in mice at the times specified. Creatine kinase (CK) and lactate dehydrogenase (LDH) levels in diluted serum samples were determined using ELISA kits (Jianglaibio, Shanghai, China) according to the manufacturer’s instructions. Enzyme Markers (BioTek Instruments, Winooski, VT, USA) were used to read the final signals.

### 4.7. Evans Blue Uptakes

The integrity of mouse muscle membranes was assessed via an EB uptake analysis. Specifically, mice were administered EB (10 mg/mL in PBS) intraperitoneally at a dosage of 0.04 mL per 10 g of body weight. This was done 18 h prior to various time points (2 h, 24 h, 48 h, 96 h) post-EIMD. Following the 18 h, the mice were euthanized, the quadriceps (Qu) muscles were harvested and sectioned using a cryostat (Leica CM 1850S). Under a fluorescence microscope, EB presented as red fluorescence. To quantify EB uptake, skeletal muscle plasma membranes (PMs) were labeled with WGA, and the percentage of EB-positive cells was determined using Image-Pro Plus 6.0 software.

### 4.8. Histology and Immunofluorescence Staining

For histological analysis, samples of the quadriceps muscle (Qu), heart, kidney, liver, and spleen from each group of mice were fixed with 4% paraformaldehyde and then embedded in paraffin. Sections of 10 μm thickness were obtained from each tissue and stained with hematoxylin solution for 20–30 min. The sections were then rinsed three times with running water and stained with eosin solution for 1–2 min. The slides were dehydrated using graded ethanol and xylene and then sealed with neutral gum for imaging.

For immunofluorescence staining, Qu muscle samples were sectioned into 10 μm slices using a Leica CM1850S cryostat microtome and then fixed in 4% PFA for 10 min. The sections were incubated with a blocking buffer for 1 h at room temperature before the primary antibodies Anti-MG53 (1/100 dilution; Abcam, Boston, MA, USA) and Xirp1 Polyclonal antibody (1/100 dilution; Thermo Fisher, Waltham, MA, USA) were added and left to incubate overnight at 4 °C. After incubation with the corresponding secondary antibody and WGA (1/200 dilution; Sigma, St. Louis, MO, USA) for 1 h at room temperature, the samples were mounted with a fluorescent medium containing DAPI for further imaging. The skeletal muscle PM was labeled with WGA. The fluorescence intensity of MG53 and the percentage of Xirp1 positive cells were quantified using Image-Pro Plus 6.0 software.

### 4.9. Skeletal Muscle’s ECM Structure as Observed by SEM

The Qu muscle was treated with 3% glutaraldehyde and then washed with 0.1 mol phosphate buffer. It was vertically fractured in liquid nitrogen and then post-fixed with 1% osmium acid in 0.1 mol phosphate buffer, followed by another wash with 0.1 mol phosphate buffer. After ethanol dehydration and isoamyl acetate replacement, the samples were subjected to critical point drying using HITACHI HCP-2 and ion sputtering using EIKO IB-3. Finally, a scanning electron microscope (HITACHI S-3400N) was used to observe any structural changes in the ECM of the skeletal muscle cross-sections.

### 4.10. Protein Extraction and Western Blot Analysis

The muscle sample was removed from the −80 °C refrigerator, weighed 100 mg before being ground to powder in a liquid nitrogen-filled mortar, and quickly transferred to a pre-chilled EP tube. An amount of 1 mL of pre-chilled RIPA lysis solution containing phosphatase, protease inhibitor, and PMSF was added, shaken, mixed, and chilled for 20 min. For testing, the supernatant was aspirated and stored at −80 °C.

The total protein content was determined using the BCA method, and the protein concentration was adjusted and heated at 70 °C for 10 min to fully denature the protein. SDS-PAGE electrophoresis was used to transfer the proteins to the PVDF membrane, which was then closed with 5% BSA at room temperature for 2 h before adding the primary antibody: PTEN (1/1000 dilution; CST), Akt (1/6000 dilution; Proteintech, Wuhan, China), pAkt (1/1000 dilution; Proteintech, Wuhan, China), MG53 (1/1000 dilution; Proteintech, Wuhan, China), MMP-2 (1/1000 dilution; Proteintech, Wuhan, China), MMP-9 (1/1000 dilution; Proteintech, Wuhan, China), TIMP1 (1/1000 dilution; Proteintech, Wuhan, China), TIMP-2 (1/1000 dilution; Proteintech, Wuhan, China), GAPDH (1/10,000 dilution; Proteintech, Wuhan, China) and shaking at room temperature for 30 min before incubating overnight at 4 °C. The PVDF membranes were washed with TBST for 3 × 10 min the next day; then a secondary antibody of appropriate concentration was added, incubated for 60 min, washed with TBST for 3 × 10 min, and developed in a dark room with ECL luminescent solution, with the appropriate exposure time determined by the fluorescence intensity. The images were analyzed semi-quantitatively using ImageJ 1.53v software after being scanned by the Bioelectrophoresis Image Analysis System.

### 4.11. Statistical Analysis

Statistical analysis was performed using the Statistical Package for the Social Sciences (SPSS 25, IBM, Armonk, NY, USA). The Shapiro–Wilks test was used to determine data normality, and the Levene test was used to ensure variance homogeneity. All data were presented in the form of mean ± standard deviation (mean ± SD). Measures based on general linear models that are two-way ANOVA were used to compare groups, and the Tukey HSD test was used for post hoc comparisons, with *p* < 0.05 considered a significant difference.

## 5. Conclusions

The current study suggests that VO promotes the recovery of motor function in EIMD mice by inhibiting both the PTEN-regulated membrane repair pathway and the ECM repair pathway.

## Figures and Tables

**Table 1:** Effect of two-week VO treatment on body weight and organ indices in mice.

Group	Heart	Left Kidney	Right Kidney	Liver	Spleen
VO	0.167 ± 0.013	0.174 ± 0.01	0.174 ± 0.011	1.469 ± 0.094	0.081 ± 0.018
VC	0.169 ± 0.01	0.173 ± 0.01	0.172 ± 0.011	1.475 ± 0.062	0.079 ± 0.018
	Pancreas	Body weight	Soleus	Gastrocnemius	Quadriceps
VO	0.155 ± 0.33	27.94 ± 2.68	0.017 ± 0.003	0.156 ± 0.013	0.239 ± 0.17
VC	0.156 ± 0.21	27.81 ± 2.65	0.016 ± 0.004	0.153 ± 0.077	0.24 ± 0.023

## Data Availability

The datasets used in the analyses described in this study are available to the corresponding author upon reasonable request.

## Supplementary Materials

The following supporting information can be downloaded at: https://www.mdpi.com/article/10.3390/ijms24129954/s1.
