# Supplementary material for: PTEN Inhibitor Treatment Lowers Muscle Plasma Membrane Damage and Enhances Muscle ECM Homeostasis after High-Intensity Eccentric Exercise in Mice
Source: Int J Mol Sci. 2023 Jun 9;24(12):9954. doi: 10.3390/ijms24129954 (PMC10298298; doi:10.3390/ijms24129954)
Supplement: Supplementary file 1 [file ijms-24-09954-s001.zip › ijms-2326974-supplementary/Figure S1.pdf]

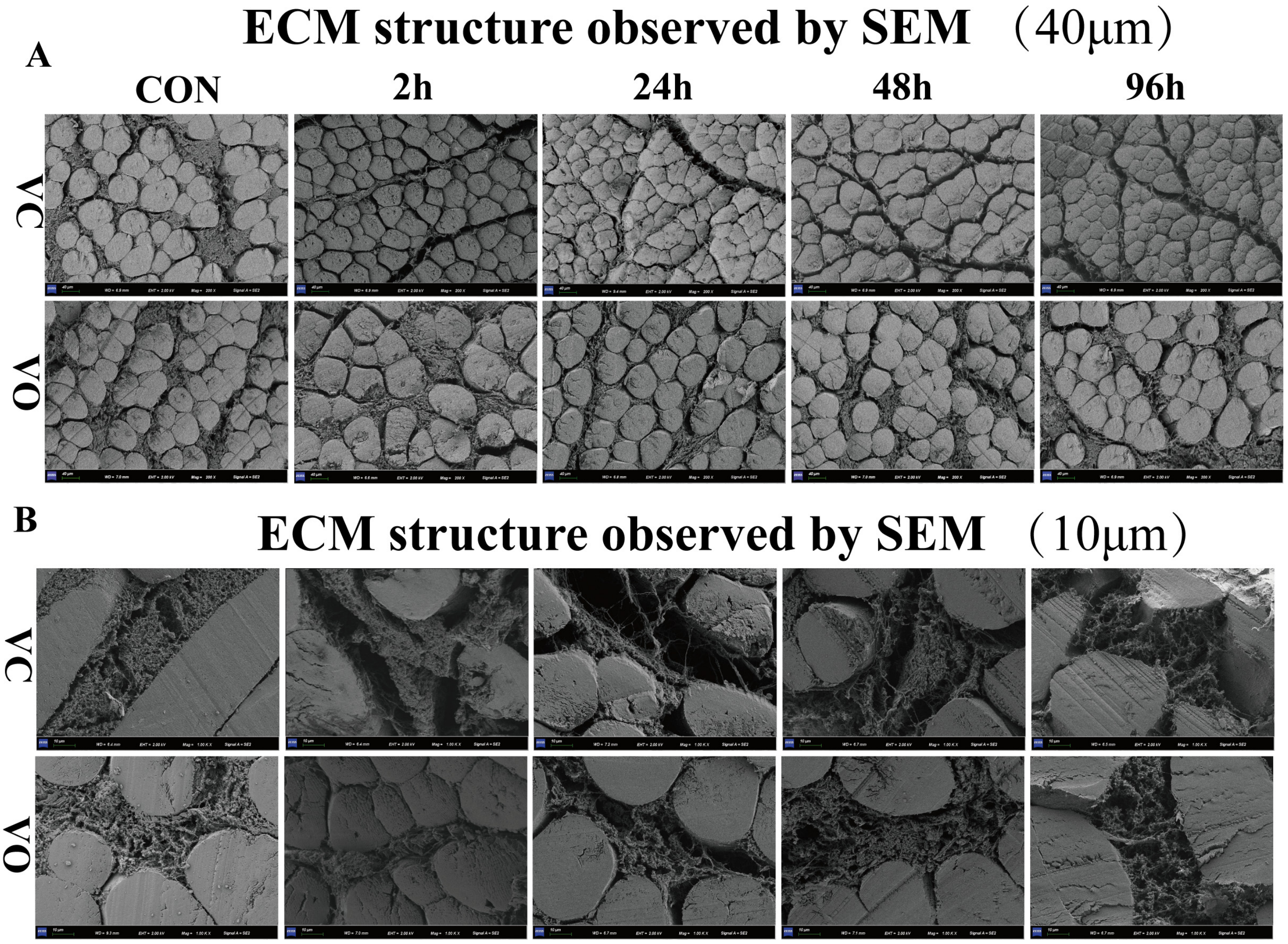

Figure S1. Representative scanning electron microscopy images of cross-sectional ECM skeletal muscle, scale bars (**A**) 40 μm (**B**) 10 μm.
